# Bioinformatic Analysis of *ABCA1* Gene Expression in Smoking and Chronic Obstructive Pulmonary Disease

**DOI:** 10.3390/membranes11090674

**Published:** 2021-08-31

**Authors:** Stanislav Kotlyarov, Anna Kotlyarova

**Affiliations:** 1 Department of Nursing, Ryazan State Medical University, 390026 Ryazan, Russia; 2 Department of Pharmacology and Pharmacy, Ryazan State Medical University, 390026 Ryazan, Russia; kaa.rz@yandex.ru

**Keywords:** ABCA1, smoking, COPD, reverse cholesterol transport, gene expression, bioinformatic analysis

## Abstract

Smoking is a key modifiable risk factor for developing the chronic obstructive pulmonary disease (COPD). When smoking, many processes, including the reverse transport of cholesterol mediated by the ATP binding cassette transporter A1 (ABCA1) protein are disrupted in the lungs. Changes in the cholesterol content in the lipid rafts of plasma membranes can modulate the function of transmembrane proteins localized in them. It is believed that this mechanism participates in increasing the inflammation in COPD. Methods: Bioinformatic analysis of datasets from Gene Expression Omnibus (GEO) was carried out. Gene expression data from datasets of alveolar macrophages and the epithelium of the respiratory tract in smokers and COPD patients compared with non-smokers were used for the analysis. To evaluate differentially expressed genes, bioinformatic analysis was performed in comparison groups using the limma package in R (v. 4.0.2), and the GEO2R and Phantasus tools (v. 1.11.0). Results: The conducted bioinformatic analysis showed changes in the expression of the *ABCA1* gene associated with smoking. In the alveolar macrophages of smokers, the expression levels of *ABCA1* were lower than in non-smokers. At the same time, in most of the airway epithelial datasets, gene expression did not show any difference between the groups of smokers and non-smokers. In addition, it was shown that the expression of *ABCA1* in the epithelial cells of the trachea and large bronchi is higher than in small bronchi. Conclusions: The conducted bioinformatic analysis showed that smoking can influence the expression of the *ABCA1* gene, thereby modulating lipid transport processes in macrophages, which are part of the mechanisms of inflammation development.

## 1. Introduction

According to rough estimates, more than a billion people smoke in the world [1,2,3]. Smoking is the main cause of the chronic obstructive pulmonary disease (COPD), the medical and social significance of which is steadily increasing [4,5]. High levels of prevalence, morbidity, and mortality of COPD carry a heavy economic burden for patients, their families, society, and the state [6,7,8,9].

According to modern concepts, the pathogenesis of COPD is based on inflammation in the bronchi, in which many cells are involved. Macrophages play an important role in the development and progression of COPD [10,11,12]. These cells are heterogeneous in their functions and demonstrate both pro- and anti-inflammatory activity, participate in the production of many humoral factors, recruit other cells. It is believed that the heterogeneity of macrophages is based on the peculiarities of their carbohydrate and lipid metabolism [13,14,15,16,17,18,19].

It is known that smoking disrupts the transport of lipids and lipid-like molecules, including cholesterol in lung cells, which may be one of the links in a complex chain of processes underlying the development and progression of COPD [20,21,22,23,24]. Cholesterol is the most important component of the plasma membranes of cells and determines their structure and function through the regulation of some transmembrane proteins. A number of recent studies indicate that reverse cholesterol transport (RCT) participates not only in ensuring the homeostasis of cellular cholesterol but also in the innate immune response [25,26]. The participation of cholesterol in the innate immune response is mediated by the ATP binding cassette transporter A1 (ABCA1) transporter that regulates RCT.

ABCA1 belongs to a large group of ATP-binding (ABC) transporters that facilitate the movement of a wide range of substrates through cell membranes. There are 48 ABC transporters in humans, which are divided into 7 subfamilies (ABCA-ABCG) based on structural characteristics. At the moment, the role of only a few ABC transporters in lung function and the development of their diseases is well known. For example, ABCA3 is involved in the formation of a surfactant, and mutations of the *ABCC7* gene (also known as cystic fibrosis transmembrane conductance regulator (CFTR)) are the cause of cystic fibrosis.

The ABCA subfamily in humans includes 12 proteins that are well known for their participation in lipid transport. ABCA1 is one of the most well-studied representatives of the ABCA subfamily. ABCA1 is expressed in various cells of many organs and it participates in the export of cholesterol and phospholipids from the cell to extracellular acceptors, thereby regulating the lipid homeostasis of cells [27,28]. Due to its role in the reverse transport of cholesterol, ABCA1 is considered an important participant in the pathogenesis of atherosclerosis. However, this is not the only known biological function of the transporter. Changing the cholesterol content in macrophages participates in the regulation of inflammation, phagocytosis, and apoptosis. ABCA1 is expressed at high levels in lung tissues and as it is believed it plays an important role in the development of COPD [29,30]. Lipid metabolism plays an important role in lung function. Moreover, the lungs are an organ with unique lipid biology. In this regard, it is interesting how smoking affects the expression of ABCA1 in various lung cells.

The purpose of this study is to analyze the patterns of expression of the human *ABCA1* gene—transporter in smoking and COPD using bioinformatics analysis methods. To do this, we use the available capabilities of modern developments that ensure the availability of biological data for repeated analysis. Similar approaches are widely used in research, including for the analysis of the differential expression of ABC transporter gene profiles in the epithelium of the respiratory tract [31].

## 2. Materials and Methods

### 2.1. Data Collection

**Figure 1:**
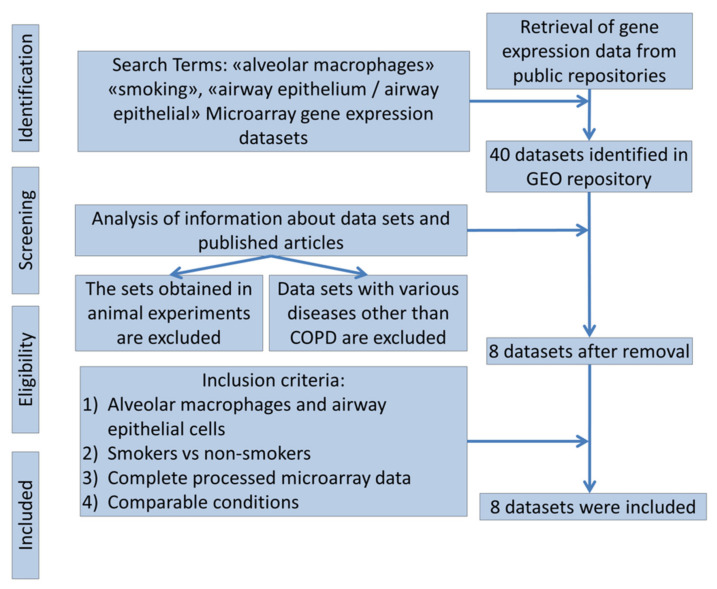
Flowcharts for microarray datasets selection.

As a data source for the analysis, publicly available sets containing information on gene expression in the airway epithelium and alveolar macrophages were used. The analysis was carried out on data sets (gene sets) obtained from The Gene Expression Omnibus (GEO), The National Center for Biotechnology Information (NCBI). The Gene Expression Omnibus (GEO) is a web database containing gene expression data and hybridization arrays, chips, microarrays (https://www.ncbi.nlm.nih.gov/geo). The search for data sets for analysis was carried out using the keywords “alveolar macrophages” “smoking”, “airway epithelium/airway epithelial” (Figure 1). 

Criteria for including sets in the analysis: (1) sets containing data on gene expression in the airway epithelium and alveolar macrophages obtained from both relatively healthy non-smokers and smokers or COPD patients; (2) comparable biomaterial sampling conditions and the presence of pre-processed gene expression data. The analysis did not include sets obtained from patients with lung cancer and other respiratory diseases, in addition to COPD, sets of experimental data obtained in animal models, as well as datasets that do not allow forming comparison groups of smokers and non-smokers. The availability of data on gene expression in COPD patients in the sets was not a prerequisite for inclusion.

According to the search criteria, the following sets were selected for analysis: GSE13896, GSE130928, GSE4498, GSE76324, GSE18385, GSE64614, GSE11906, GSE11784 (Table 1).

GSE13896 contained data on gene expression in alveolar macrophages obtained during bronchoalveolar lavage in 24 healthy non-smokers, 34 smokers, and 12 smokers with COPD [32]. The data was obtained using the GPL570 platform [HG-U133_Plus_2] Affymetrix Human Genome U133 Plus 2.0 Array

GSE130928 contained data on gene expression in alveolar macrophages obtained during bronchoalveolar lavage in 24 healthy non-smokers, 42 smokers, and 22 smokers with COPD [33]. The data was obtained using the GPL570 platform [HG-U133_Plus_2] Affymetrix Human Genome U133 Plus 2.0 Array.

GSE4498 contained data on gene expression in the bronchial epithelium in 10 phenotypically normal smokers compared with 12 non-smokers [34,35]. The data were obtained using the GPL570 platform [HG-U133_Plus_2] Affymetrix Human Genome U133 Plus 2.0 Array.

Data on gene expression in the 3rd–4th-order bronchial epithelium obtained in 20 healthy non-smokers and 31 healthy smokers, and in the 10th–12th-order bronchial epithelium obtained in 57 healthy non-smokers and 52 healthy smokers were included in the analysis from the GSE76324 set [36,37]. The data were obtained using the GPL570 platform [HG-U133_Plus_2] Affymetrix Human Genome U133 Plus 2.0 Array.

GSE18385 contained data on gene expression in the epithelium of the bronchi of 3-4 orders, obtained in 21 healthy non-smokers, 31 healthy smokers, and small respiratory tract (10th–12th orders of the bronchi) in 51 healthy non-smokers, 58 healthy smokers, obtained by bronchoscopy [38]. The data was obtained using the GPL570 platform [HG-U133_Plus_2] Affymetrix Human Genome U133 Plus 2.0 Array.

Data on gene expression in the bronchial epithelium, including the trachea [n = 27] and bronchi of the 4–6th order [n = 20] obtained in healthy non-smokers and the epithelium of the distal respiratory tract (bronchi of the 10th–12th order) obtained in 44 healthy non-smokers and 36 healthy smokers were included in the analysis from the GSE64614 set [39]. The data was obtained using the GPL570 platform [HG-U133_Plus_2] Affymetrix Human Genome U133 Plus 2.0 Array.

Data on gene expression in samples of the tracheal epithelium, bronchi of the 2nd–3rd order, and bronchi of the 10–12th order, selected by fiber-optic bronchoscopy in 124 people (42 healthy non-smokers, 49 healthy smokers and 33 smokers with chronic respiratory symptoms and smokers with COPD) were included in the analysis from the GSE11906 set [40]. The data was obtained using the GPL570 platform [HG-U133_Plus_2] Affymetrix Human Genome U133 Plus 2.0 Array.

Data on gene expression in the bronchial epithelium of the 10th–12th order obtained in 63 healthy non-smokers, 72 healthy smokers, and 22 patients with COPD were included in the analysis from the GSE11784 set [41,42]. The data was obtained using the GPL570 platform [HG-U133_Plus_2] Affymetrix Human Genome U133 Plus 2.0 Array.

The datasets selected for analysis were obtained from different studies and differed in data normalization methods. GSE13896, GSE4498, GSE76324, GSE18385, GSE64614, GSE11906, GSE11784, used the MAS5 normalization method, where GSE130928 used the Robust Multi-array Average (RMA) method. The sets were analyzed in accordance with the methods of obtaining and normalizing data that were used in the original study. Data from different sets were analyzed independently of each other and were not combined for analysis.

### 2.2. Data Extraction

For each dataset, the following information was extracted: the platform, the number of smokers, COPD patients and healthy non-smokers, smoking experience (pack-years index), the location in the respiratory tract from which samples were obtained (bronchial generation, trachea) and pre-processed gene expression data. To analyze the data on the expression of the *ABCA1* gene, comparison groups were formed: smokers, healthy individuals, and patients with COPD (Table 2). Table 2 shows the demographic and clinical characteristics of the patients whose data make up the sets selected for analysis sets (GSE13896, GSE130928, GSE4498, GSE18385, GSE11906, GSE11784), except for the sets GSE76324, GSE64614, which do not contain information about patients.

In addition to the smoking status and the presence of COPD, data on smoking intensity (the pack-years index) were also taken into account. Other available categorical data from the sets were not analyzed in this study.

### 2.3. Differential Expression Analysis

In this study, the analysis of the differential expression of the *ABCA1* gene in each of the sets in the comparison groups was carried out using GEO2R, Phantasus (v. 1.11.0), and the limma package in R (v. 4.0.2).

GEO2 (http://www.ncbi.nlm.nih.gov/geo/geo2r/) is an interactive web tool for the analysis to compare gene expression levels in groups in a dataset of GEO. Phantasus (https://artyomovlab.wustl.edu/phantasus/)—a web application for visual and interactive gene expression analysis.

Using these tools, data on the differential expression of the *ABCA1* gene in the comparison groups for each set, including *p*-value, logFC, were obtained. If necessary, log2 transformation and quantile normalization was performed. To adjust the level of statistical significance during multiple comparisons the algorithm of Benjamini& Hochberg (FDR-false discovery rate) was used, implemented in GEO2R, and using the limma package and the p. adjust function in R (v. 4.0.2) [43]. All *p* values satisfying the condition < 0.05 at FDR ≤ 0.1 were taken as statistically significant.

Visualization of the *ABCA1* gene expression levels in the comparison groups in each of the data sets was carried out using the Phantasus tool (v. 1.11.0) [44]. The data is visualized as box diagrams. The diagrams visualize the minimum value (lower part of the vertical line), the first-third quartile (box), the median (horizontal line inside the box), and the maximum value (upper part of the vertical line) of the data distribution.

## 3. Results

**Figure 2:**
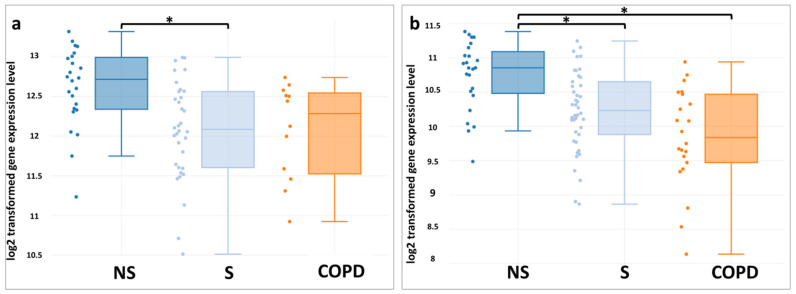
Patterns of expression of the *ABCA1* gene in alveolar macrophages (**a**—GSE13896; **b**—GSE130928) associated with smoking status: between healthy non-smokers (NS, dark blue color) and smokers without a COPD diagnosis (S, light blue color) and with a COPD diagnosis (COPD, orange color). All datasets were generated from the Affymetrix Human Genome U133 Plus 2 microarray platform. GSE13896 used the Mas 5.0 Normalization method and GSE130928 used the RMA Normalization method. The data distribution is visualized in the form of box diagrams. Statistically significant differences (*p* values and *p* values adjusted using the algorithm of Benjamini & Hochberg) are shown with asterisks: * *p* < 0.01, FDR ≤ 5%.

The conducted bioinformatic analysis of the data sets of alveolar macrophages (GSE13896 and GSE130928) showed that smoking alters the expression of the ABCA1 gene. A statistically significant decrease in the expression levels of ABCA1 in the alveolar macrophages of smokers compared with non-smokers is determined (Figure 2). No differences in the expression of ABCA1 were found in smokers and patients with COPD. At the same time, a downregulated expression of the gene in COPD patients was also marked compared with non-smokers, which was found in the GSE130928 set (Figure 2b).

The obtained results correspond to the available data that cigarette smoke suppresses the RCT in alveolar macrophages, mediated by the ABCA1 transporter. Alveolar macrophages are important participants in inflammation in COPD [45,46]. These unique cells are in constant contact with inhaled microorganisms and exogenous particles and provide participation as the first line of the body defense. Macrophages are not homogeneous in their origin and participation in the pathogenesis of COPD. They play an important role not only in the implementation of the innate immune response but also perform a number of regulatory functions, participate in apoptosis [45,46].

**Figure 3:**
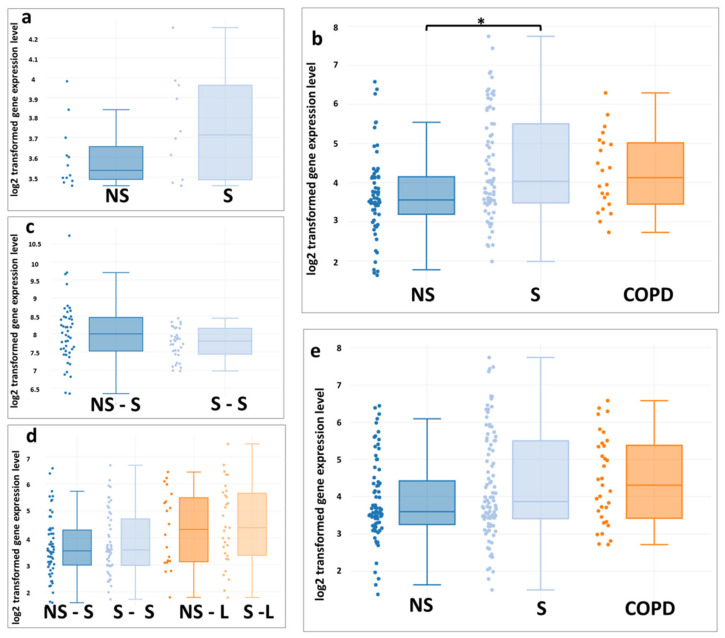
Patterns of *ABCA1* expression in the respiratory tract epithelium (**a**—GSE4498; **b**—GSE11784; **c**—GSE64614; **d**—GSE76324; **e**—GSE11906) associated with smoking status: non-smokers (NS) non-smokers–small airways (NS–S); non-smokers–large airways (NS–L); smokers without a diagnosis of COPD (S); smokers–large airways (S–L); smokers–small airways (S–S) and with a diagnosis of COPD (COPD). All datasets were generated from the Affymetrix Human Genome U133 Plus 2 microarray platform (using the Mas 5.0 Normalization method). The data distribution is visualized in the form of box diagrams. Statistically significant differences (*p* values and *p* values adjusted using the algorithm of Benjamini & Hochberg) are shown with asterisks: * *p* < 0.01, FDR ≤ 5%.

The analysis of ABCA1 gene expression in the epithelium of the respiratory tract in smokers showed contradictory results. No statistically significant changes in the expression of ABCA1 were found in the sets GSE4498, GSE11906, GSE64614, GSE76324, and GSE18385, whereas in the set GSE11784 the expression of the ABCA1 gene was upregulated in smokers (more than 10 pack-years) (Figure 3).

**Figure 4:**
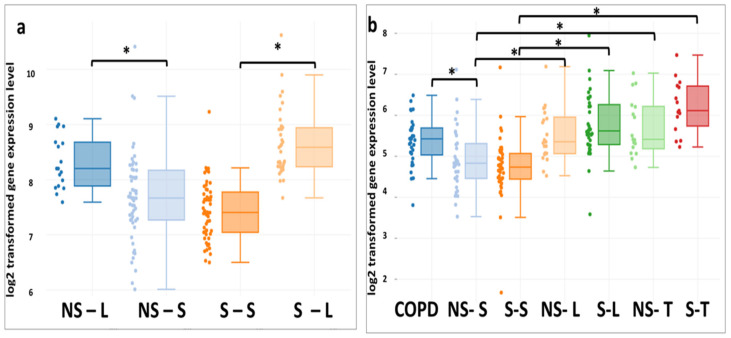
Patterns of *ABCA1* expression in the epithelium of the upper and lower respiratory tract (**a**—GSE18385; **b**—GSE11906) associated with smoking status: non-smokers–large airways (NS–L), non-smokers–small airways (NS–S), smokers–large airways (S–L), smokers–small airways (S–S), non-smokers–trachea (NS–T), non-smokers–trachea (NS–T), diagnosis of COPD–small airways (COPD). All datasets were generated from the Affymetrix Human Genome U133 Plus 2 microarray platform (using the Mas 5.0 Normalization method). The data distribution is visualized in the form of box diagrams. Statistically significant differences (*p* values and *p* values adjusted using the algorithm of Benjamini & Hochberg) are shown with asterisks: * *p* < 0.01, FDR ≤ 5%.

It was also found that both smokers and non-smokers have upregulated expression of ABCA1 in the epithelial cells of the large bronchi (generation 2nd–4th) than small (generation 10th–12th) ones (Figure 4).

In general, the obtained results indicate that cigarette smoking, which is a modifiable risk factor for the development of COPD, is associated with differentiated patterns of the ABCA1 gene expression.

## 4. Discussion

We conducted a bioinformatic analysis of the ABCA1 gene expression in alveolar macrophages and airway epithelium in smokers, non-smokers, and COPD patients from GEO datasets and showed that the levels of gene expression in the alveolar macrophages of smokers are lower than in non-smokers. These data may indicate the effect of smoking on the expression of ABCA1.

Our experimental project included the analysis of publicly available databases obtained from alveolar macrophages (GSE13896, GSE130928) and respiratory tract epithelium (sets GSE4498, GSE76324, GSE18385, GSE64614, GSE11906, GSE11784) to determine the differential expression of the ABCA1 gene associated with smoking. Bioinformatic analysis of gene expression data placed in publicly available databases is widely used in research both for evaluating differentially expressed genes and for their functional analysis.

Using tools for online analysis, we formed comparison groups: smokers, healthy non-smokers, and patients with COPD. Statistically significant differences in gene expression levels were taken into account, which was corrected in accordance with the algorithm of Benjamini & Hochberg.

In a previous study using similar tools, the authors analyzed the differential expression of ABC transporter genes in the epithelium of the respiratory tract during smoking, in patients with COPD and bronchial asthma [31]. We analyzed the differential expression of one representative of a large family of ABC transporters—ABCA1 to confirm the information about the effect of smoking on it.

Literature data suggest that smoking has a significant effect on the expression and function of ABCA1 in the respiratory tract. The data obtained in recent years have expanded our understanding of the function of the ABCA1 protein [30]. This representative of a large family of ABC transporters is a key participant in the formation of high-density lipoprotein (HDL) due to its ability to export cholesterol and phospholipids from the cell to the extracellular acceptor. In this regard, the role of ABCA1 is well known in the pathogenesis of atherosclerosis [47]. However, taking into account the high levels of ABCA1 expression in lung tissues, it becomes obvious that the function of the transporter is much more extensive than it was thought previously [48,49,50]. The significance of Abca1 for lung function is well demonstrated by experimental data with gene knockout in mice that develop pronounced morphological changes in the lungs that increase with age and are characterized by the accumulation of foamy macrophages, destruction of alveolar septa, and epithelization of the alveoli due to severe hypertrophy and hyperplasia of type II pneumocytes [51]. The described morphological changes were accompanied by a decrease in tidal volume and hyperventilation [52].

The expression of ABCA1 is influenced by many factors. The transporter has a complex transcriptional and post-transcriptional regulation, which involves both cholesterol metabolites and humoral inflammatory factors [53,54,55,56,57,58].

The choice of alveolar macrophages for analysis is due to the multifaceted role of ABCA1 in the function of these cells. It is known that lung macrophages act as the first line of immune defense of the lungs. These cells are heterogeneous in their origin and functions. They play an important role in the pathogenesis of COPD and their number increases significantly in the lungs with COPD. ABCA1 is involved in providing several functions of macrophages associated with inflammation [59,60]. The activity of phagocytosis by macrophages may be associated with the levels of expression and functional activity of ABCA1, which ensures the removal of excess cholesterol engulfed during phagocytosis. Conversely, there is a decrease in the phagocytic activity of ABCA1 deficient macrophages. A decrease in the expression and functional activity of the transporter leads to a decrease in the RCT and its excessive accumulation in macrophages, which has great consequences for their inflammatory activation [26,61]. Cholesterol can directly act as a trigger for the cellular inflammatory response and affect some signaling pathways.

An important mechanism that ensures the participation of ABCA1 in inflammation is the transporter-mediated regulation of the cholesterol content in the lipid rafts of plasma membranes [62,63]. Lipid rafts are specialized membrane microdomains of the plasma membrane of cells, enriched with cholesterol and sphingolipids. The structure of lipid rafts is dynamic, which is associated with the constantly changing content of both lipids and proteins. Cholesterol is the most important component of lipid rafts, as it is necessary for their formation and configuration [64]. Moreover, cholesterol performs not only a structural role, due to the rigid sterane backbone. It is believed that it is able to interact directly with the transmembrane domains of proteins and influence their activity [65]. ABCA1, by changing the cholesterol level, ensures the stability of lipid rafts and leads to the activation or deactivation of related proteins, for example, Toll-like receptor TLR4, which regulates the inflammatory response to lipopolysaccharide (LPS) of Gram-negative bacteria and plays an important role in the pathogenesis of COPD. Upon activation, TLR4 is localized in lipid rafts and their destruction disrupts the signal transduction of the receptor [66].

A decrease in the expression of ABCA1 and its functional activity during smoking leads to a decrease in RCT and intracellular accumulation of cholesterol, which contributes to the activation of inflammation through several mechanisms [26,61,67]. Conversely, an increase in the functional activity of ABCA1 can have an anti-inflammatory effect by removing excess cholesterol [48,68,69].

Thus, a decrease in the expression of ABCA1 caused by smoking can lead to the inflammatory activation of macrophages. The data obtained in this study indicate that smoking is associated with a decrease in the expression of ABCA1 in alveolar macrophages.

Cholesterol transport is not the only function of ABCA1, since it is involved in the movement of other lipids that are, for example, part of a surfactant [70]. This role is well demonstrated by a study with the accumulation of vacuoles in type II pneumocytes in mice with a knockout of the Abca1 gene, which indicates insufficient surfactant secretion [52]. Thus, the role of ABCA1 in macrophages and alveolar epithelial cells may be different.

The analysis of the expression of ABCA1 in the epithelium of large and small airways showed that there are variations in the results depending on which data set is being studied. In most datasets (GSE4498, GSE11906, GSE64614, GSE76324, and GSE18385), there were no statistically significant differences in the expression of ABCA1 in the comparison groups, whereas, in the GSE11784 set, the expression of the ABCA1 gene was increased in smokers. When interpreting these data, differences in the expression of ABCA1 in different types of bronchial epithelial cells should be taken into account [71]. However, the data sets did not differentiate cell types. In addition, in some sets, differences were found in the expression of ABCA1 in the epithelial cells of the trachea and large bronchi, compared with small airways. In a previous study, similar expression dynamics were demonstrated for another representative of the ABCA subfamily, ABCA13, whose function is not completely clear, but it is also believed to be involved in lipid transport [31]. These data may indicate that there are differences in lipid transport in different parts of the respiratory tract.

Previous studies have already shown a violation of ABCA1 expression in smoking and COPD [59,60,72]. The data obtained in this study confirm the available data. It should be noted that COPD is a heterogeneous disease with various clinical manifestations, which are based on the features of pathophysiological mechanisms, many of which are not clear today. Taking into account the large heterogeneity of COPD patients and the fact that different lung cells with differences in lipid metabolism may be involved in the pathogenesis of COPD in different ways, more data are needed to interpret and understand violations of the expression and functional activity of ABCA1 in smoking and COPD. It is also important that many other factors besides smoking, including bacterial colonization of the bronchi, can affect lipid metabolism and lipid transport processes in COPD [73]. However, in general, the data accumulated to date indicate the important role of lipids located at the intersection of many signaling pathways in providing immune protection of the lungs.

It should be noted that the present study has some limitations due to the fact that the data sets contain a small number of patients; there is not enough information about the patients taking medications that can affect lipid metabolism. However, these limitations, typical for bioinformatic analysis, may be useful for planning further experimental research.

In this regard, it is interesting to further study the role of the ABCA1 transporter in different cells of the respiratory tract, as well as in cells with different functional activity. Bioinformatic analysis is a useful tool that can be used to analyze data to obtain new information on gene expression, as well as when planning experimental studies.

## 5. Conclusions

Thus, the conducted bioinformatic analysis showed that smoking can influence the expression of the *ABCA1* gene, thereby modulating lipid transport processes in macrophages and epithelium of the respiratory tract, which are part of the mechanisms of inflammation development.

## Figures and Tables

**Table 1:** Characteristics of the analyzed GEO datasets.

DataSet	Characteristic	Method of Obtaining the Material	Microarray Platform	References
GSE13896	Alveolar macrophages obtained from healthy non-smokers, smokers, and smokers with COPD	Bronchoalveolar lavage	GPL570 Affymetrix Human Genome U133 Plus 2.0 Array	[32]
GSE130928	Alveolar macrophages obtained from healthy non-smokers, smokers, and smokers with COPD	Bronchoalveolar lavage	GPL570 Affymetrix Human Genome U133 Plus 2.0 Array	[33]
GSE4498	The bronchial epithelium of the 10th–12th order obtained in healthy non-smokers and phenotypically normal smokers	Bronchoscopy	GPL570 Affymetrix Human Genome U133 Plus 2.0 Array	[34,35]
GSE76324	Bronchial epithelium of the 3rd–4th and 10th–12th order, obtained in healthy non-smokers and smokers	Bronchoscopy	GPL570 Affymetrix Human Genome U133 Plus 2.0 Array	[36,37]
GSE18385	Bronchial epithelium of the 3rd–4th and 10th–12th order, obtained in healthy non-smokers and smokers	Bronchoscopy	GPL570 Affymetrix Human Genome U133 Plus 2.0 Array	[38]
GSE64614	Bronchial epithelium, including trachea and bronchi of the 4th–6th order obtained from healthy non-smokers and epithelium of the distal respiratory tract (bronchi of the 10th–12th order) of healthy non-smokers and healthy smokers	Bronchoscopy	GPL570 Affymetrix Human Genome U133 Plus 2.0 Array	[39]
GSE11906	Bronchial epithelium of trachea, bronchi of the 2nd–3rd order, and bronchi of the 10th–12th order obtained from healthy non-smokers, smokers, and smokers with COPD	Bronchoscopy	GPL570 Affymetrix Human Genome U133 Plus 2.0 Array	[40]
GSE11784	Bronchial epithelium of the 10th–12th order obtained in healthy non-smokers, smokers, and patients with COPD	Bronchoscopy	GPL570 Affymetrix Human Genome U133 Plus 2.0 Array	[41,42]

**Table 2:** Demographic and clinical characteristics of patients in GEO datasets.

DataSet	Characteristic	Comparison Groups		
		Non-Smokers	Smokers	COPD
GSE13896	Age (year) Pack-years index Sex (M/F)	40.2 ± 8.3 - 18/6	42.1 ± 7.8 27.5 ± 18.1 25/9	54.2 ± 9.3 51.5 ± 29.2 10/2
GSE130928	Age (year) Pack-years index Sex (M/F)	40.3 ± 8.2 - 18/6	42.6 ± 7.8 27.1 ± 17.0 29/13	53.8 ± 7.9 43.4 ± 28.0 18/4
GSE4498	Age (year) Pack-years index Sex (M/F)	42.3 ± 7.7 - 10/2	44.0 ± 3.9 25.8 ± 9.1 7/3	-
GSE18385	Age (year) Pack-years index Sex (M/F)	41.1 ± 10.5 - 51/21	43.1 ± 7.0 28.1 ± 17.2 59 / 30	-
GSE11906	Age (year) Pack-years index Sex (M/F)	42.6 ± 9.7 - 32/10	42.9 ± 6.3 26.9 ± 15.9 35/14	55.6 ± 7.7 36.4 ± 21.9 26/7
GSE11784	Age (year) Pack-years index Sex (M/F)	40.3 ± 12 - 40/23	42.5 ± 7.6 27.2 ± 15.9 52/20	51.6 ± 8.6 40.9 ± 28.2 18/4

## Data Availability

The data presented in this study are available on request from the corresponding author.
